# Mulinane- and Azorellane-Type Diterpenoids: A Systematic Review of Their Biosynthesis, Chemistry, and Pharmacology

**DOI:** 10.3390/biom10091333

**Published:** 2020-09-17

**Authors:** Angel de Jesús Dzul-Beh, Andrés Humberto Uc-Cachón, Jorge Bórquez, Luis A. Loyola, Luis Manuel Peña-Rodríguez, Gloria María Molina-Salinas

**Affiliations:** 1 Unidad de Investigación Médica Yucatán, Unidad Médica de Alta Especialidad Hospital de Especialidades 1 Mérida, Yucatán, Instituto Mexicano del Seguro Social, Mérida 97150, Yucatán, Mexico; angeldzulbeh1992@gmail.com (A.d.J.D.-B.); andresuccachon@gmail.com (A.H.U.-C.); 2 Departamento de Química, Facultad de Ciencias Básicas, Universidad de Antofagasta, Antofagasta 02800, Chile; jorge.borquez@uantof.cl (J.B.); aloyola@uantof.cl (L.A.L.); 3 Unidad de Biotecnología, Centro de Investigación Científica de Yucatán, A.C., Mérida 97205, Yucatán, Mexico; lmanuel@cicy.mx

**Keywords:** mulinane, azorellane, diterpenoids, antimycobacterial, antiulcer

## Abstract

Mulinane- and azorellane-type diterpenoids have unique tricyclic fused five-, six-, and seven-membered systems and a wide range of biological properties, including antimicrobial, antiprotozoal, spermicidal, gastroprotective, and anti-inflammatory, among others. These secondary metabolites are exclusive constituents of medicinal plants belonging to the *Azorella*, *Laretia*, and *Mulinum* genera. In the last 30 years, more than 95 mulinanes and azorellanes have been reported, 49 of them being natural products, 4 synthetics, and the rest semisynthetic and biotransformed derivatives. This systematic review highlights the biosynthetic origin, the chemistry, and the pharmacological activities of this remarkably interesting group of diterpenoids.

## 1. Introduction

For millennia, humankind has relied heavily on nature to provide for its basic needs, and to alleviate a wide spectrum of diseases. It is well documented that plants constitute the basis of traditional medicine systems; fossil records date the human use of plants as medicines to at least the Middle Paleolithic age, some 60,000 year ago [1,2]. In Mesopotamia, the uses of approximately 1000 plant-derived substances were documented around 2600 B.C. [3]. Currently, herbal remedies continue to be used for the treatment of different diseases by a large number of people; it has been reported that between 70% and 95% of the population, mainly in developing countries, still use traditional medicine as their primary health care when caring for their health-related needs and concerns [4].

Many plant species have been reported to possess pharmacological activities which are due to their content of natural products, broadly defined as small molecules derived from primary metabolites (e.g. carbohydrates, amino acids, etc.), used by the plant to mediate its interactions with the surrounding environment [5,6]. These natural products are genetically encoded and are produced by secondary metabolic pathways [6]. The four main families of secondary metabolites include polyketides, terpenoids, polyphenols derivatives, and alkaloids, and can be found in the leaves, stems, root, and bark of plants [7].

Since the early 20th century, and to date, natural products have received a great deal of attention because of their importance in the development of new pharmaceuticals [7]. It has been reported that between 50 to 70% of the approved small-molecule drugs that came into market in the period between 1981 to 2014 were derived from natural products, including unaltered natural products, chemically modified derivatives, and synthetic natural mimics derived from a natural-product template or a pharmacophore [8]. In addition, natural products and their derivatives represent more than one-third of all U.S. Federal Drug Administration (FDA)-approved new molecular entities, especially for antibiotic and anticancer molecules [9].

**Figure 1:**
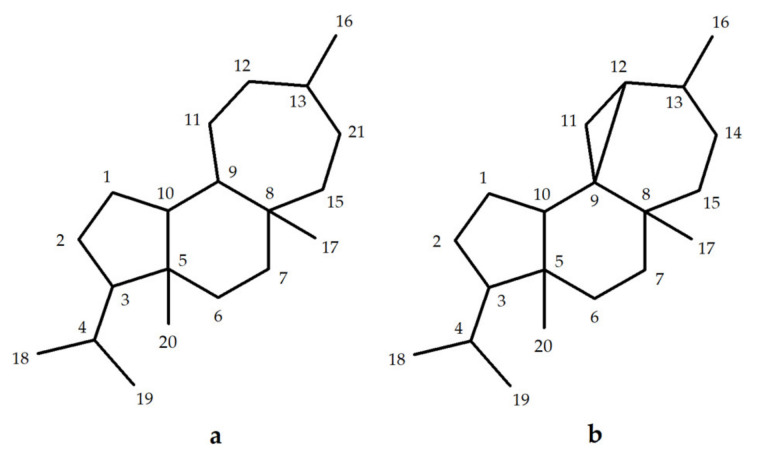
Carbon skeletons of mulinane (**a**) and azorellane (**b**) diterpenoids.

The mulinane and azorellane diterpenoids are a group of structurally-interesting natural products which have been reported to show a wide variety of biological activities including antimicrobial, antiprotozoal, antitumor, anti-inflammatory, and antimycobacterial, among others [10]. While mulinanes have a tricyclic skeleton of fused five-, six-, and seven-membered rings, with an angular substituent at each of the ring junctions, the skeleton of the azorellanes includes a tetracyclic arrangement (Figure 1). Nearly all mulinanes are characterized by having a carboxyl group at the C-20 position and a functionalized seven-member ring. In contrast, in azorellanes C-20 is not functionalized but C-13 is usually oxygenated [11,12].

These diterpenoids have been reported exclusively from species of the genus *Azorella*, *Laretia* and *Mulinum* (Apiaceae, Umbelliferae) [10], and particularly from *Azorella compacta* Phil and *Mulinum crassifolium* Phil. In fact, the analysis of *A. compacta* resin by gas chromatography-mass spectrometry showed that the relative composition was 99% diterpenoids, where the dominant components were oxygenated diterpenoids [13]. Both species have traditionally been used by native people in South America for their medicinal properties; *A. compacta* is reportedly used for the treatment of different types of colds, as well as bronchitis, asthma, inflammation, diabetes, skin disorders, toothache, backache, and disorders of the kidney and uterus [14,15], and *M. crassifolium* is used to treat diabetes, bronchial and intestinal disorders, and stomach problems [15,16].

This review provides an outlook on the biosynthesis, chemistry, and pharmacological properties of mulinane and azorellane diterpenoids.

## 2. Literature Search

A precise literature search was carried out with Google Scholar, Scopus, PubMed, Science Direct, CONRICYT repositories for related findings. The following keywords: Activity + Mulinum + Diterpenoids, Activity + Laretia + Diterpenoids, Activity + Azorella + Diterpenoids, Diterpenoids + Azorella, Diterpenoids + Laretia, Diterpenoids + Mulinum, Diterpenoids + Azorellane, Diterpenoids + Mulinane, Activity + Diterpenoids + Azorellane, Activity + Diterpenoids + Mulinane, were used to find all the relevant literature published on mulinanes and azorellanes, their biosynthesis, sources, pharmacological activities, synthesis, and modifications conferred on their structure. In this research, time interval was not used, all related findings were included.

## 3. Mulinane and Azorellane Biosynthesis

**Figure 2:**
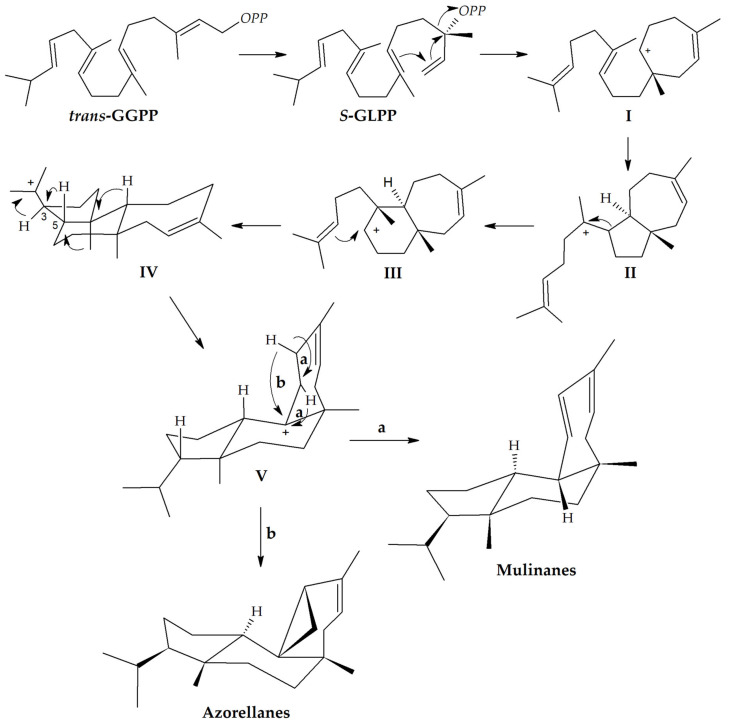
Biosynthetic pathway to mulinane and azorellane diterpenoid skeletons. GGPP: geranylgeranyl pyrophosphate; *S*-GLPP: *S*-geranyllinaloyl pyrophosphate; OPP: Pyrophosphate [11].

While it has been suggested that mulinane biogenesis derives from the biogenetic transformation of a labdane derivative [17], a different proposal for the biosynthetic origin of mulinane and azorellane diterpenoids (Figure 2) starts with the cyclization of the C-20 general precursor geranylgeranyl pyrophosphate (GGPP) to produce all *trans*-GGPP, which is then isomerized to *S*-geranyllinaloyl pyrophosphate (S-GLPP); an anti-Markovnikov cyclization generates the enantiomeric cation I that, by means of cyclization, yields the bicyclic system II a sigmatropic rearrangement, and leads to the stereoisomeric ion III having angular methyl groups considered the *syn* precursor of mulinanes and azorellanes. Cyclization of the side chain yields the tricyclic system IV, with an all-trans ring junctions’ stereochemistry, that, following a series of 1,2-hydride and methyl shifts, leads to V. The intermediate V is the true precursor of mulinanes and azorellanes; a 1,2-hydride shift followed by deprotonation yields the mulinane skeleton, while the loss of the allylic proton produces the cyclopropane ring found in azorellanes [11].

## 4. Sources and Chemical Structures of Natural Mulinane and Azorellane Diterpenoids

Species of the genera *Mulinum*, *Azorella*, and *Laretia* are well-recognized sources of diterpenoids with mulinane and azorellane skeletons. While mulinanes are only present in *Mulinum* spp., the *Azorella* and *Laretia* genera are known to produce secondary metabolites with both mulinane and azorellane skeletons [10]. *Azorella* spp., *Mulinum* spp., and *Laretia* spp. include perennial shrubs, cushions, or mat-forming species that are adapted to cold and windy terrain and are often found at high-elevation habitats, particularly in the Andes mountain range of South America [18]. These species are distributed in southernmost South America and in the Subantarctic islands, as well as south of Australia and New Zealand. In South America, they extend from the Subantarctic region northward through the Patagonian steppes of Argentina and Chile and further north, they are restricted to the Andes Plateau highlands [19].

**Figure 3:**
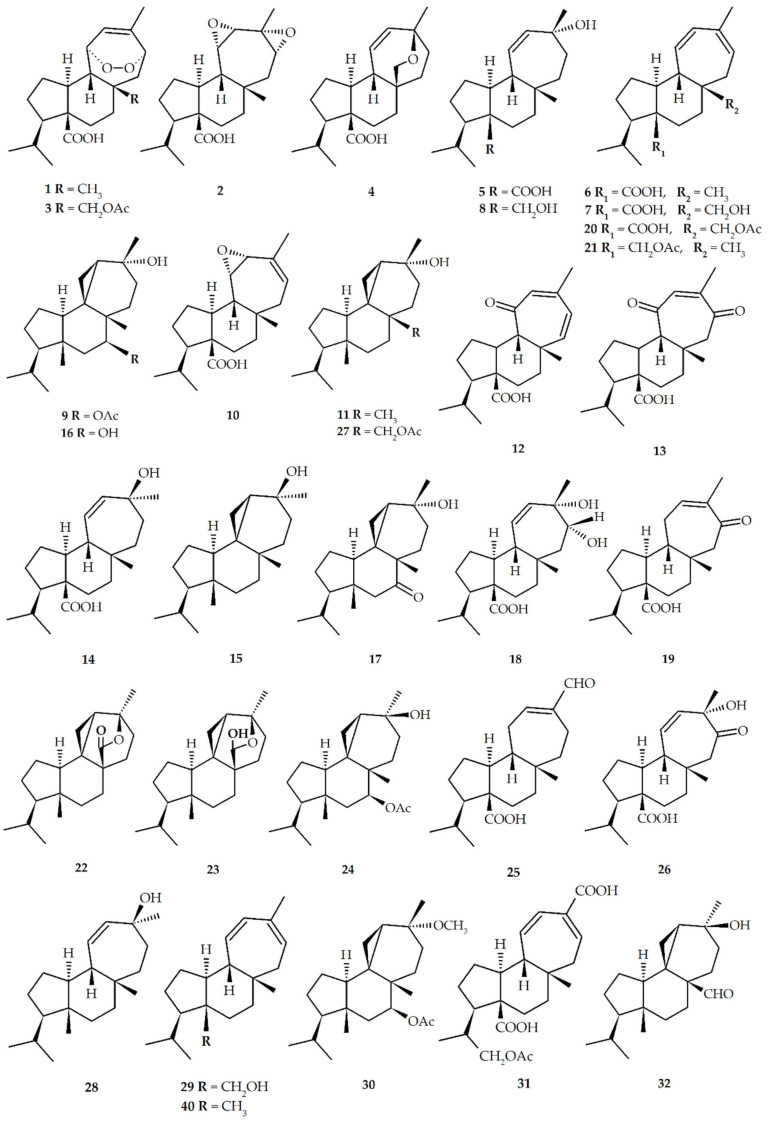

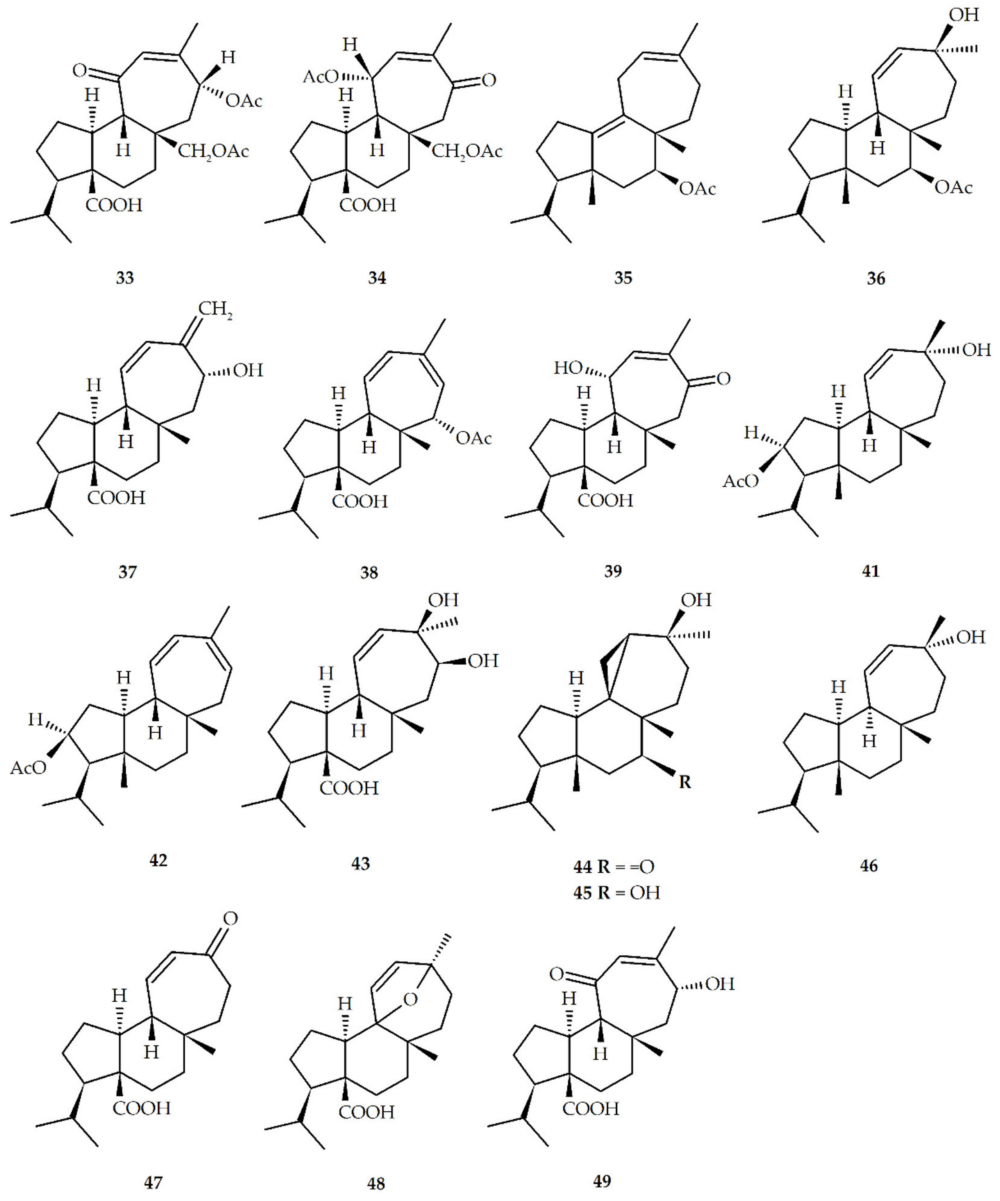
Natural mulinane and azorellane diterpenoids.

The first mulinane diterpenoids, mulinic acid (**1**) and isomulinic acid (**2**), were reported from *M. crassifolium* in 1990 [17]; similarly, the first azorellane diterpenoid was azorellanol (**9**) from *A. compacta* in 1998 [20]. To date, 37 mulinane and 12 azorellane diterpenoids have been isolated from *Azorella* spp., *Laretia* spp., and *Mulinum* spp. (Table 1, and Figure 3).

## 5. Synthetic, Semisynthetic, and Biotransformed Mulinane and Azorellane Diterpenoids

**Figure 4:**
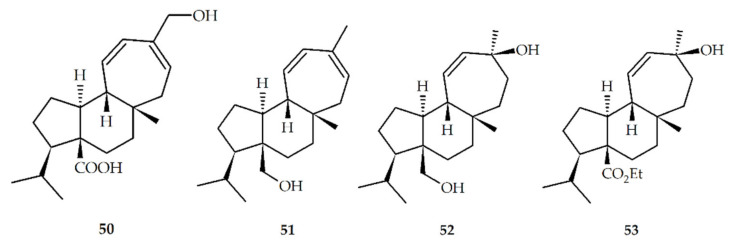
Synthetic mulinanes diterpenoids.

**Figure 5:**
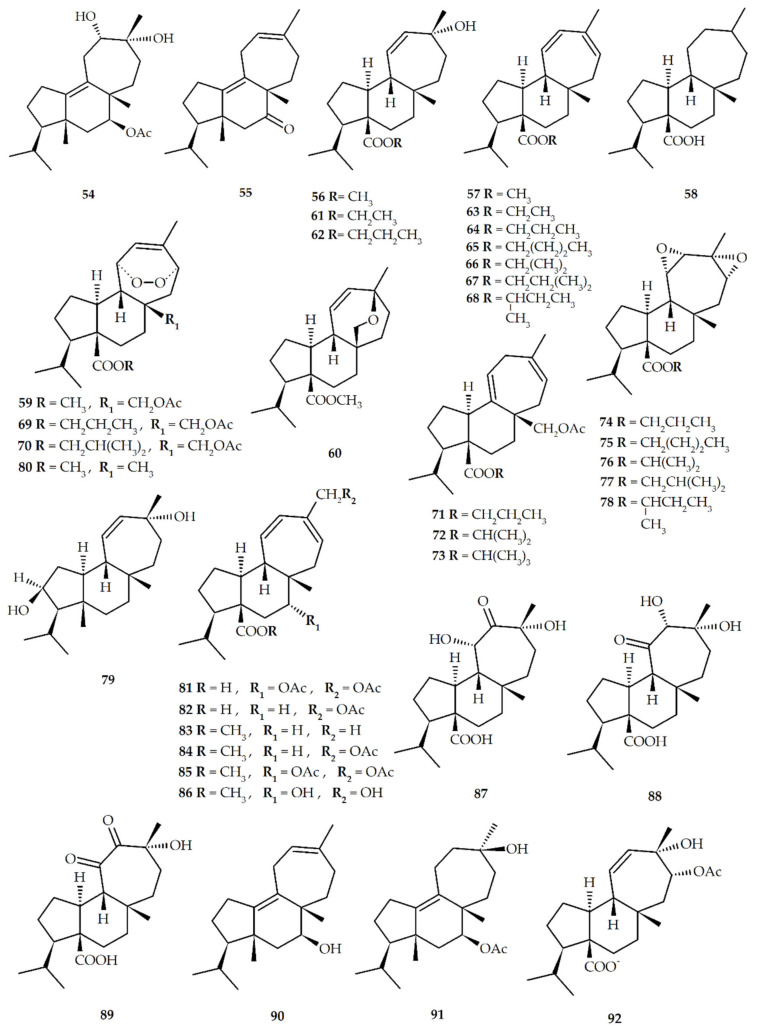
Semisynthetic mulinane diterpenoids.

**Figure 6:**
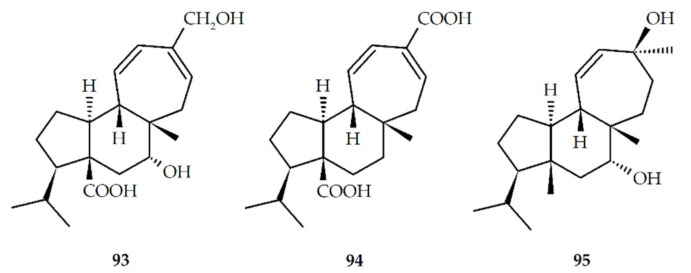
Biotransformed mulinane diterpenoids.

Recently, Liu et al. achieved the enantioselective total synthesis of natural mulinanes and analogous from cyclopentenone (Table 2, and Figure 4) [53]. In addition, the presence of different functional groups, which include hydroxyl, carboxyl, acetoxy, and double bond, in the mulinane skeleton has allowed the preparation of a significant number of semisynthetic derivatives using dehydration, alkylation, hydrolysis, and oxidation reactions (Table 3, and Figure 5). To date, only one semisynthetic azorellane derivative, 7*β*-deacetylazorellanol (**16**), has been reported; this can be explained by the fact that the cyclopropane ring in the azorellane skeleton can be easily open under weak acidic conditions, to produce semisynthetic derivatives having a mulinane skeleton [54]. However, the limited number of functionalized positions, and the type of functional groups, found in the chemical structures of natural azorellane and mulinane diterpenoids limit the number and type of semisynthetic derivatives that can be prepared through chemical modification [55]; because of this, in recent years, biotransformation using filamentous fungi such as *Mucor plumbeus* and *M. circinelloides* has been explored as a new strategy to obtain novel mulinane and azorellane derivatives [51,55,56] (Table 4, Figure 6).

## 6. Pharmacological Activities of Mulinane and Azorellane Diterpenoids

Despite the development of drugs for treating diseases such as HIV/AIDS, malaria, tuberculosis, hypertension, diabetes, and cancer, these diseases continue to affect diverse populations worldwide with significant associated mortalities and the need to develop new and more effective pharmaceuticals is always present [61]. Currently, the importance of natural products and/or its derivatives in drug discovery and development is well recognized [8,62]. Since the structurally-unique mulinanes and azorellanes have displayed a wide variety of biological activities in both in vitro and in vivo pharmacological models, the preparation of mulinane and azorellane derivatives could yield new products with a stronger biological activity, a better solubility, or useful in determining structure-activity relationships or mode-of-action [63]. A summary of the pharmacological activities reported for mulinanes and azorellanes is listed in Table 5.

### 6.1. Antimicrobial Activity

Since the initial discovery of antibiotics in the 1930s, and perhaps until the 1980s, the prevailing view was that virtually all bacterial infections could be treated effectively with antibiotics. The spread of antimicrobial resistance has generated a growing concern in the medical community and in the public. The rate of persons dying due to the lack of effective antimicrobials is growing; it has been estimated that the number of deaths occurring worldwide due to drug resistance could rise to as many as 10 million individuals in 2050 [62]. Hence, the development of new antibiotics is needed. A number of mulinane and azorellane diterpenoids have displayed significant antimycobacterial and antibacterial activities, e.g., azorellan-13*β*-ol (**15**) from *Azorella madreporica* showed activity against *Mycobacterium tuberculosis* (H37Rv) with a minimal inhibitory concentration (MIC) = 20 µg/mL in the BD BACTEC MGIT 960 system [46]. Natural mulinanes and azorellanes isolated from *A. compacta*, *A. madreporica*, *M. crassifolium*, and *Laretia acaulis*, together with a number of semisynthetic derivatives, were evaluated against susceptible *M. tuberculosis* (H37Rv) and MDR (resistant to Streptomycin, Isoniazid, Rifampin, Ethambutol, and Pyrazinamide) clinical isolate using the Microplate Blue Alamar Assay (MABA). The natural mulin-11-en-13*α*,20-diol (**8**), azorellanol (**9**), 13*β*-hydroxyazorellane (**15**), azorellanone (**17**), 17-acetoxy-13-*α*-hydroxyazorellane (**27**), semisynthetic 13*α*-hydroxy-mulin-11-en-20-oic-acid methyl ester (**56**), mulin-11,13-dien-20-oic acid methyl ester (**57**), and mulinenic acid methyl ester (**60**) were the most active against both strains (MIC = 12.5–25 µg/mL) [54,57], taking into account that a MIC of ≤64 µg/mL is considered promising for a pure product [64]. Preparation of several C-20 alkylated mulinane derivatives confirmed reports that methylation of the C-20 carboxyl group of the mulinane skeleton improves activity [59]; the most active derivatives, with MIC values between 6.25 and 25 µg/mL against the drug-sensitive and drug-resistant strains of *M. tuberculosis*, included 13*α*-hydroxy-mulin-11-en-20-oic acid *n*-propyl-ester (**62**), the isomulinic acid *n*-propyl ester (**74**), and the isomulinic acid *n*-butyl ester (**75**). The results also demonstrated that linear esters of mulinanes had better antituberculosis activity than their branched counterparts [59]. Similarly, the semisynthetic derivative 7*β*-acetoxy-mulin-9,12-diene (**35**) showed antimycobacterial activity (MIC = 43.8 µg/mL) when tested against *M. smegmatis*, as well as antimicrobial activity against *Staphylococcus aureus* (ATCC 25923) and MDR (resistant to Ampicillin-sulbactam, Cefoxitin, Cephalotin, Cephazolin, Ciprofloxacin, Clindamycin, Erythromycin, and Trimethoprim-sulfamethoxazole) strains [22]. Other metabolites, such as mulin-12,14-dien-11-on-20-oic acid (**12**) and mulin-12-ene-11,14-dion-20-oic acid (**13**), both isolated from *A. compacta*, exhibited activity against Methicillin-resistant and -susceptible strains of *S. aureus*, as well as Vancomycin-resistant and -susceptible strains of *Enterococcus faecium* and *Escherichia coli* [40].

### 6.2. Antiprotozoal Activity

Parasitic diseases are a serious health problem that has had a deep impact on the global human population [65]. Among parasites, protozoal parasites such as *Trypanosoma cruzi*, *Leishmania* spp., *Plasmodium falciparum*, *Giardia intestinalis*, *Trichomonas vaginalis*, and *Toxoplasma gondii*, represent the major disease-causing organisms [65,66]. The infections caused by these parasites are responsible for 500 million deaths worldwide, especially in undeveloped countries, where a tropical or temperate climate and poor sanitary and hygiene conditions are common [67]. Globally, the burden of protozoal diseases is increasing and has been exacerbated by the limited number of pharmaceuticals available, the lack of effective medication due to drug resistance, the severity of side effects, the high costs, or their limited practicality for field use. These limitations have prompted many researchers to search for novel drugs against protozoal parasites [65,67]. Diverse studies provide support that mulinanes and azorellane represent a promising group of natural antiprotozoal agents [21,29,35,41]. Azorellanol (**9**) and mulin-11,13-dien-20-oic acid (**6**), both isolated from *A. compacta*, showed strong in vitro trypanocidal activity (IC_50_ values of 20–87 µM) when tested against epimastigotes, trypomastigotes, and amastigotes of different strains (Tulahuen, SPA-14, and CL Brener) of *T. cruzi*. Both metabolites also showed activity against intracellular amastigotes of the CL Brener clone with an IC_50_ of 32.3 µM and 29 µM, respectively [21]. Additionally, azorellanol (**9**) also had an effect on trophozoites of *T. vaginalis* Ant-1 strain (LD_50_ = 40.5 mM) and *T. gondii* (ID_50_ = 54 mM), but 7*β*-deacetylazorellanol (16) showed a stronger activity (ID_50_ = 42 mM) against *T. gondii* [35,41]. Finally, 17-acetoxymulin-11,13-dien-20-oic acid (**20**) and 13*α*,14*α*-dihydroxymulin-11-en-20-oic acid (**18**), both from *A. compacta*, caused 60% and 42% growth inhibition of *Plasmodium berghei* NK 65 in infected mice, respectively, when tested at a dose of 10 mg/kg/day [29].

### 6.3. Spermicidal/Spermatostatic Activity

Mulinane and azorellane diterpenoids have been evaluated in terms of several parameters that characterize human sperm function, i.e., sperm motility and viability, sperm binding to the human zona pellucida, the progesterone-induced acrosome reaction, an increase in intracellular Ca^2+^ concentration, and protease activity in the search for a contraceptive method to inhibit, in a reversible and specific manner, the functions of the male gamete. Azorellanone (**17**), isolated from *A. yareta*, inhibited sperm motility in a concentration-dependent manner (0.15‒3 mM), while sperm viability was inhibited at 3 mM. Assays with 17 significantly inhibited sperm–zona binding, progesterone-induced acrosome reactions, and intracellular Ca^2+^ concentration. Additionally, **17** also affected protease activity and inhibited trypsin- and chymotrypsin-like activities. These results suggest that **17** may be a potential candidate as a contraceptive agent used in the manufacture of vaginal jellies or creams [68]. Other diterpenoids, such as mulinenic acid (**4**), mulinolic acid (**5**), and azorellan-17,13-(*β*)olide (**22**), have been evaluated for their spermatostatic activity. Compounds (**5)** and (**22)** demonstrated significant spermatostatic properties [31].

### 6.4. Antidiabetic

Mulinolic acid (**5**) and azorellanol (**9**), both isolated from *A. compacta*, were evaluated for their antidiabetic activity in Streptozotocin-induced diabetic rats and both metabolites decreased glycemia at 180 mg/mL, a similar value to that observed for Chlorpropamide used as positive control. Azorellanol (**9**) increased the insulin levels in serum; however, with (**5**) the levels of insulin remained unchanged; these results suggested that while (**9)** could be acting on the β-cells of pancreatic islets, (**5**) may be acting on glucose utilization or production in the liver [23].

### 6.5. Antiulcer

A number of azorellanes (**9**, **11**, and **15**) and mulinanes (**1**, **5**, **6**, **28**, **29**, **31**, and **40**) isolated from *A. compacta* exhibited gastroprotective activity in HCl/EtOH-induced gastric lesions in mice, at a dose of 20 mg/kg. The best activity was caused by mulin-11,13-dien-18-acetoxy-16,20-dioic acid (**31**), azorellanol (**9**), and 13*β*-hydroxyazorellane (**15**), with values (73–69%) similar to those observed for the positive control Lansoprazole (78–68%) at the same dose [24,26]. Similarly, mulin-11,13-dien-20-oic acid (**6**) isolated from *A. trifurcata* also demonstrated a gastroprotective effect (ED_50_ = 55 mg/kg); the possible mode or gastroprotective action of **6** was evaluated in mice using a pre-treatment with various blockers such as Indomethacin (inhibitor of prostaglandin synthesis), N-ethylmaleimide (blocker of sulfhydryl compounds), N-nitro-L-arginine methyl ester (inhibitor of nitric oxide), and ruthenium red (vanilloid receptor antagonist). The results suggested that prostaglandins and sulfhydryl compounds are positively involved in the gastroprotective activity of this metabolite [36]. The gastroprotective effect of mulinanes has led to the exploration of different chemical modifications in the diterpenoid structure to improve activity; the evaluation of a number of semisynthetic (**81**–**86**) and biotransformed (**50**,**93**) mulinane derivatives have allowed the identification of 7*α*,16-dihydroxymulin-11,13-dien-20-oic acid (**93**, 69%) and 16-hydroxy-mulin-11,13-dien-20-oic acid (**50**, 59%) as the most active derivatives when tested at a dose of 20 mg/kg, both showing a better gastroprotective effect than Lansoprazole (57%) at the same dose. Interestingly, an apparent relationship between the polarity of the derivatives and their gastroprotective activity was established, with the decrease in polarity causing the loss of activity [51].

### 6.6. Anti-Inflammatory

A search for bioactive metabolites with anti-inflammatory and analgesic properties produced by *A. compacta*, *A. yareta*, and *L. acaulis* resulted in the identification of azorellanol (**9**), azorellanone (**17**), and 7*β*-deacetylazorellanol (**16**); azorellanol (**9**) showed anti-inflammatory activity when tested on arachidonic acid (AA) and 12-deoxyphorbol-13-Tetradecanoate (TPA)-induced edemas. The fact that (**9)** showed a higher activity on the TPA than in the AA-induced edema assays (dose: 15 × 10–7 mol/ear) and that the dermal anti-inflammatory activity was of 70.8%, suggests that the mechanism of action of (**9)** could involve the inhibition of cyclo-oxygenase activity. Alternatively, (**17)** showed the strongest analgesic activity when the three metabolites were tested in the acetic acid-induced abdominal constriction response in mice model (59% of analgesic effect, dose: 10 × 10^–5^ mol/kg) [42]. Additional studies on the anti-inflammatory properties of azorellanol (**9**) showed its having an effect (25 mg/mL) on the inhibition of the transcription factor Nuclear Factor-kappa Beta (NF-κB), one of the key regulators of the genes involved in the immune/inflammatory response [70], in the NF-κB-dependent luciferase gene reporter assay. Finally, 7*β*-deacetylazorellanol (**16**) also demonstrated anti-NF-κB activity [44].

### 6.7. Cytotoxic

Other activities reported for mulinanes and azorellanes include cytotoxic activity on human breast adenocarcinoma cells (MCF-7); mulin-11,13-dien-20-oic acid (6), azorellanol (9), 7*β*-deacetylazorellanol (16), 13*β*-epiazorellanol (24), and 7*β*-acetoxy-mulin-9,12-diene (35) displayed good cytotoxic activity (less than 50% cell viability at 100 µM), with azorellanol (9) being the most active (IC_50_ = 25.64 µM). The results obtained in this investigation suggested that the beta acetate group at C-7 was required to increase the cytotoxic effect. Additionally, the alpha position of the OH group at position C-13 in the same skeleton further increased the cytotoxic effect when comparing the cytotoxic effect of the epimers 16 (OH-13 in beta position) and 9 (OH-13 in alpha position) [28].

### 6.8. Anti-Alzheimer

Mulinanes have also been evaluated in the inhibition of acetylcholinesterase (AChE). Inhibition of AChE serves as a strategy for the treatment of Alzheimer disease (AD), senile dementia, ataxia, myasthenia gravis, and Parkinson disease, and it has been considered as a potential therapeutic approach to AD. Mulinolic acid (5) and mulin-11,13-dien-20-oic acid (6), both isolated from *A. trifurcata*, have shown moderate inhibitory activity toward the enzyme AChE in a colorimetric assay with IC_50_ of 200 and 180 µg/mL, respectively [69].

## 7. Conclusions

This is a report that systematically describes the biosynthesis, occurrence, isolation, structures, and biological activities of mulinane and azorellane diterpenoids. In summary, a total of 95 of these compounds has been reported since 1990. Thirty-seven mulinanes and 12 azorellanes have been isolated from species of *Azorella*, *Laretia*, and *Mulinum* genera. Synthesis, chemical modifications, and biotransformation by *Mucor plumbeus* and *M. circinelloides* have produced 4 synthetics and 44 mulinane derivatives. Even though these diterpenoids have been extensively studied because of their biological properties such as antimicrobial, antiprotozoal, spermicidal, gastroprotective, anti-inflammatory, antidiabetic, cytotoxic, and anti-Alzheimer, a large number of mulinanes and azorellanes have shown important anti-*M. tuberculosis* and gastroprotective activities. The antimycobacterial activity of the semisynthetic *n*-propyl (**74**) and *n*-butyl (**75**) esters of isomulinic acid (**2**) and of 13*α*-hydroxy-mulin-11-en-20-oic-acid *n*-propyl ester (**62**) suggest that an increase in the size/length of the substituent could increase the potency of mulinane derivatives.

Traditional uses of species of *Azorella*, *Laretia*, and *Mulinum* for stomach ulcers led to the evaluation of the diterpenoid compounds isolated from these plants in gastroprotective model. Mulin-11,13-dien-18-acetoxy-16,20-dioic acid (**31**), azorellanol (**9**), and 13*β*-hydroxyazorellane (**15**) demonstrated best gastroprotective effect. The biotransformation and chemical modification of mulin-11,13-dien-20-oic acid (**6**) led to the improvement of its gastroprotective effect.

Currently, only a small amount of research has been involved in the analysis of the structure‒activity relationship in mulinanes and azorellanes. Similarly, studies on the target genes, target proteins, and signaling pathways involved in the mechanisms of action of mulinane and azorellane diterpenoids are limited; these studies are necessary in order have a mulinane or azorellane become a potential pharmaceutical. With this review we intended to make a significant contribution to the current knowledge about these interesting diterpenoids, as well as to encourage their continuing study.

## Figures and Tables

**Table 1:** Mulinane and azorellane diterpenoids isolated from *Mulinum* spp., *Azorella* spp., and *Laretia* spp.

Structure No.	Diterpenoid	Skeleton Type	First Report	Species	References
**1**	Mulinic acid (mulin-11,14-peroxi-12-en-20-oic acid)	M	1990	*M. crassifolium* *A. compacta* *A. trifoliata*	[17,21,22,23,24,25,26]
**2**	Isomulinic acid (mulin-11,12,13,14-diepoxy-20-oic acid)	M	1990	*M. crassifolium*	[17]
**3**	17-acetoxymulinic acid (mulin-17-acetoxy-11,14-peroxi-12-en-20-oic acid)	M	1990	*M. crassifolium* *A. compacta*	[10,16,27,28,29]
**4**	Mulinenic acid (mulin-13*α*-hydroxy-11-en-20-oic acid	M	1991	*M. crassifolium* *A. compacta* *M. spinosum*	[30,31,32]
**5**	Mulinolic acid (mulin-13*α*-hydroxy-11-en-20-oic acid)	M	1996	*M. crassifolium* *L. acualis* *A. yareta* *A. compacta* *A. madreporica* *M. spinosum* *A. trifoliata*	[10,16,21,23,24,26,28,29,31,32,33,34,35,36,37,38,39,40]
**6**	Mulin-11,13-dien-20-oic acid	M	1996	*M. spinosum* *A. compacta* *A. yareta* *M. crassifolium* *L. acaulis* *A. trifurcata* *A. cuatrecasasii* *M. spinosum*	[16,21,23,24,26,28,33,35,36,37,38]
**7**	17-hydroxy-mulin-11,13-dien-20-oic acid (mulin-17-hydroxy-11,13-dien-20-oic acid)	M	1996	*M. spinosum*	[37]
**8**	Mulinol (mulin-11-en-13*α*,20-diol)	M	1997	*A. compacta* *A. criptantha*	[25,41,42,43]
**9**	Azorellanol (azorellan-13*α*-hydroxy-7*β*-yl acetate)	A	1998	*A. compacta* *A. yareta* *L. acaulis* *A. trifurcata* *A. criptantha*	[10,20,21,22,23,24,26,28,35,36,37,41,42,44]
**10**	11,12-epoxy-mulin-13-en-20-oic acid (mulin-11,12-epoxy-13-en-20-oic acid)	M	1998	*A. compacta*	[45]
**11**	13α-hydroxy-azorellane (azorellan-13α-ol)	A	1998	*A. madreporica* *A. yareta* *A. compacta* *A. trifurcata* *A. trifoliata*	[22,24,26,35,36,39,46,47]
**12**	Mulin-12,14-dien-11-on-20-oic acid	M	1999	*A. compacta*	[40]
**13**	Mulin-12-ene-11,14-dion-20oic acid	M	1999	*A. compacta*	[40]
**14**	13*β*-epimulinolic acid (mulin-13*β*-hydroxy-11-en-20-oic acid)	M	2000	*L. acaulis*	[38]
**15**	13*β*-hydroxyazorellane (azorellan-13*β*-ol)	A	2001	*A. yareta* *A. compacta* *L. acaulis* *A. trifurcata*	[24,35,36,44,46]
**16**	7*β*-deacetylazorellanol (azorellan-13*α*,7*β*-diol)	A	2001	*L. acaulis* *A. compacta* *A. trifoliata*	[21,22,23,28,41,42,44]
**17**	Azorellanone (azorellan-13*α*-hydroxy-7-one)	A	2003	*A. yareta* *A. trifurcata* *L. acaulis*	[42]
**18**	13α,14α-dihydroxymulin-11-en-20-oic acid (mulin-13*α*,14*α*-dihydroxy-11-ene-20-oic acid)	M	2003	*M. spinosum* *A. compacta*	[28,29,32]
**19**	14-oxo-mulin-12-en-20-oic acid (mulin-12-en-14-oxo-20-oic acid)	M	2003	*M. spinosum*	[32]
**20**	17-acetoxy-mulin-11,13-dien-20-oic acid (mulin-17-acetoxy-11,13-dien-20-oic acid)	M	2004	*A. compacta*	[28,29]
**21**	20-hydroxymulin-11,13-dienyl acetate (mulin-11,13-dien-20-yl acetate)	M	2004	*A. compacta*	[29]
**22**	Azorellolide (azorellan-17,13-(*β*)olide)	A	2004	*A. cryptantha* *M. spinosum* *L. acaulis*	[31,43,48]
**23**	Dyhydroazorellolide (azorellan-17,13-(*β*)hemiacetal)	A	2004	*A. cryptantha*	[48]
**24**	13*β*-epiazorellanol (azorellan-13*β*-hydroxy-7*β*-yl acetate)	A	2007	*L. acaulis* *A. compacta*	[28,44]
**25**	Mulin-12-en-16-al-20-oic acid	M	2010	*A. madreporica*	[39]
**26**	13*α*-hydroxy-mulin-11-en-14-one-20-oic acid (mulin-13*α*-hydroxy-14-oxo-11-en-20 oic acid)	M	2010	*A. madreporica*	[39]
**27**	17-acetoxy-13*α*-hydroxyazorellane (azorellan-13*α*-hydroxy-17-yl acetate)	A	2011	*A. madreporica*	[47]
**28**	13*β*-hydroxymulinane (mulin-13*β*-ol)	M	2013	*A. compacta*	[24]
**29**	Mulin-11,13-dien-20-ol	M	2013	*A. compacta*	[24]
**30**	13*α*-methoxyazorellanol (azorellan-13*α*-methoxy-7*β*-yl acetate)	M	2013	*A. compacta*	[24]
**31**	Mulin-11,13-dien-18-acetoxy-16,20-dioic acid	M	2013	*A. compacta* *A. trifurcata*	[24,36]
**32**	Azorelaldehyde (azorellan-13*α*-hydroxy-17-al)	A	2014	*A. cryptantha*	[49]
**33**	Mulinone A (mulin-14*α*,17-diacetoxy-12-en-11-oxo-20-oic acid)	M	2014	*M. crassifolium*	[10]
**34**	Mulinone B (mulin-11α,17-diacetoxy-12-en-14-oxo-20-oic acid)	M	2014	*M. crassifolium*	[10]
**35**	7-acetoxy-mulin-9,12-diene (mulin-9,12-dien-7*β*-yl acetate)	M	2014	*M. crassifolium* *A. compacta*	[10,28]
**36**	7*α*-acetoxy-9-epi-13*β*-hydroxymulinane (mulin-9-epi-13*β*-hydroxy-11-en-7*β*-yl acetate)	M	2014	*A. trifurcata*	[36]
**37**	14*α*-hydroxymulin-11,13(16)-dien-20-oic acid (mulin-14*α*-hydroxy-11,13(16)-dien-20-oic acid)	M	2014	*A. trifurcata*	[36]
**38**	15*α*-acetoxymulin-11,13-dien-20-oic acid (mulin-15*α*-acetoxy-11,13-dien-20-oic acid)	M	2014	*A. trifurcata*	[36]
**39**	11*α*-hydroxymulin12-en-14-one-20-oic acid (mulin-11*α*-hydroxy-12-en-14-oxo-20-oic-acid)	M	2014	*A. trifurcata*	[36]
**40**	Mulin-11,13-diene	M	2014	*A. compacta*	[26]
**41**	2-acetoxy-13*α*-hydroxy-mulin-11-ene (mulin-13*α*-hydroxy-11-en-2-yl acetate)	M	2014	*A. spinosa*	[50]
**42**	2-acetoxy-mulin-11,13-diene (mulin-11,13-dien-2-yl acetate)	M	2014	*A. spinosa*	[50]
**43**	13*β*,14*β*-dihydroxymulin-11-en-20-oic acid (mulin-13*β*,14*β*-dihydroxy-11-en-20-oic acid)	M	2015	*A. compacta*	[28]
**44**	13*β*-epiazorellanone (azorellan-13*β*-hydroxy-7-one)	A	2015	*A. compacta*	[28]
**45**	13*β*-epi-7*β*-deacetyl-azorellanol (azorellan-13*β*,7*β*-diol)	A	2015	*A. compacta*	[28]
**46**	9-epi-13*α*-hydroxymulinene (mulin-9-epi-13*α*-ol)	M	2016	*A. cuatrecasassi*	[51]
**47**	Normulin-11-en-13-oxo-20-oic acid	M	2018	*A. compacta*	[52]
**48**	9,13-epoxymulin-11-en-20-oic acid (mulin-9,13-epoxy-11-en-20-oic acid)	M	2019	*M. crassifolium*	[16]
**49**	14*α*-hydroxymulin-12-en-11-one-20-oic acid (mulin-14*α*-hydroxy-12-en-11-oxo-20-oic acid)	M	2019	*M. crassifolium*	[16]

M: Mulinane; A: Azorellane.

**Table 2:** Synthetic derivatives of mulinanes [53].

No	Synthetic Mulinanes
1 *	Mulinic acid (mulin-11,14-peroxi-12-en-20-oic acid)
2 *	Isomulinic acid (mulin-11,12,13,14-diepoxy-20-oic acid)
6 *	mulin11,13-dien-20-oic acid
14 *	13*β*-epimulinolic acid (mulin-13*β*-hydroxy-11-en-20-oic acid)
21 *	20-hydroxymulin-11,13-dienyl acetate (mulin-11,13-dien-20-yl acetate)
**50**	16-hydroxy-mulin-11,13-dien-20-oic acid (mulin-16-hydroxy-11,13-dien-20-oic acid)
**51**	Mulin-11,13-dien-20-ol
**52**	13*β*-epi-mulinol (mulin-11-en-13*β*,20-diol)
**53**	13*β*-epi-mulinolic acid ethyl ester (mulin-13*β*-hydroxy-11-en-20-oic acid ethyl ester)

* Previously reported as natural product.

**Table 3:** Semisynthetic derivatives of mulinanes.

No.	Semisynthetic Mulinanes	References
35 *	7*β*-acetoxy-mulin-9,12-diene (mulin-9,12-dien-7*β*-yl acetate)	[22,57,58]
**54**	7*β*-acetoxy-12*α*,13*α*-dihydroxy-mulin-9-ene (mulin-12*α*,13*α*-dihydroxy-9-en-7*β*-yl acetate)	[54,57]
**55**	7-oxo-mulin-9,12-diene (mulin-9,12-dien-7-one)	
**56**	13*α*--hydroxy-mulin-11-en-20-oic-acid methyl ester (mulin-13*α*-hydroxy-11-en-20-oic acid methyl ester)	
**57**	Mulin-11,13-dien-20-oic acid methyl ester	
**58**	Mulin-20-oic acid	
**59**	17-acetoxy-mulinic acid methyl ester (mulin-17-acetoxy-11,14-peroxi-12-en-20-oic acid methyl ester)	
**60**	Mulinenic acid methyl ester (mulin-13*α*,17-oxy-11-en-20-oic acid methyl ester)	
**61**	13*α*--hydroxy-mulin-11-en-20-oic-acid ethyl ester(mulin-13*α* -hydroxy-11-en-20-oic acid ethyl ester)	[54,59]
**62**	13*α*--hydroxy-mulin-11-en-20-oic-acid *n*-propyl ester (mulin-13*α* -hydroxy-11-en-20-oic acid *n*-propyl ester)	
**63**	Mulin-11,13-dien-20-oic acid ethyl ester	
**64**	Mulin-11,13-dien-20-oic acid *n*-propyl ester	
**65**	Mulin-11,13-dien-20-oic acid *n*-butyl ester	
**66**	Mulin-11,13-dien-20-oic acid *iso*-propyl ester	
**67**	Mulin-11,13-dien-20-oic acid *iso*-butyl ester	
**68**	Mulin-11,13-dien-20-oic acid *sec*-butyl ester	
**69**	17-acetoxy-mulinic acid *n*-propyl ester (mulin-17-acetoxy-11,14-peroxi-12-en-20-oic acid *n*-propyl ester)	
**70**	17-acetoxy-mulinic acid *iso*-butyl ester (mulin-17-acetoxy-11,14-peroxi-12-en-20-oic acid *iso*-butyl ester)	
**71**	17-acetoxy-mulin-9(11),13(14)-dien-20-oic acid *n*-propyl ester (mulin-17-acetoxy-9(11),13(14)-dien-20-oic acid *n*-propyl ester)	
**72**	17-acetoxy-mulin-9(11),13(14)-dien-20-oic acid *iso*-propyl ester (mulin-17-acetoxy-9(11),13(14)-dien-20-oic acid *iso*-propyl ester)	
**73**	17-acetoxy-mulin-9(11),13(14)-dien-20-oic acid *sec*-butyl ester (mulin-17-acetoxy-9(11),13(14)-dien-20-oic acid *sec*-butyl ester)	
**74**	Isomulinic acid *n*-propyl ester (mulin-11,12,13,14-diepoxy-20-oic acid *n*-propyl ester)	
**75**	Isomulinic acid *n*-butyl ester (mulin-11,12,13,14-diepoxy-20-oic acid *n*-butyl ester)	
**76**	Isomulinic acid *iso*-propyl ester (mulin-11,12,13,14-diepoxy-20-oic acid *iso*-propyl ester)	
**77**	Isomulinic acid *iso*-butyl ester (mulin-11,12,13,14-diepoxy-20-oic acid *iso*-butyl ester)	
**78**	Isomulinic acid *sec*-butyl ester (mulin-11,12,13,14-diepoxy-20-oic acid *sec*-butyl ester)	
**79**	2,13*α*-dihydroxymulin-11-ene (mulin-11-en-2,13*α*-diol)	[50]
**80**	Mulinic acid methyl ester (mulin-11,14-peroxi-12-en-20-oic acid methyl ester)	[22]
**81**	7*α*,16-diacetoxy-11,13-dien-20-oic acid (mulin-7α,16-diacetoxy-11,13-dien-20-oic acid)	[51]
**82**	16-acetoxymulin-11,13-dien-20-oic acid (mulin-16-acetoxy-11,13-dien-20-oic acid)	
**83**	16-hydroxymulin-11,13-dien-20-oic acid methyl ester (mulin-16-hydroxy-11,13-dien-20-oic acid methyl ester)	
**84**	16-acetoxymulin-11,13-dien-20-oic acid methyl ester (mulin-16-acetoxy-11,13-dien-20-oic-acid methyl ester)	
**85**	7*α*,16-dihydroxymulin-11,13-dien-20-oic acid methyl ester (mulin-7*α*,16-dihydroxy-11,13-dien-20-oic acid methyl ester)	
**86**	7*α*,16-diacetoxymulin-11,13-dien-20-oic acid methyl ester (mulin-7*α*,16-acetoxy-11,13-dien-20-oic acid methyl ester)	
**87**	12-oxo-11*α*,13*α*-dihydroxymulin-20-oic acid (mulin-11*α*,13*α*-dihydroxy-12-oxo-20-oic acid)	[58]
**88**	11-oxo-12*α*,13*α*-dihydroxymulin-20-oic acid (mulin-12*α*,13*α*-dihydroxy-11-oxo-20-oic acid)	
**89**	11,12-dioxo-13α-hydroxymulin-20-oic acid (mulin-13*α*-hydroxy-11,12-dioxo-20-oic acid)	
**90**	Mulin-9,12-dien-7-ol	
**91**	7*β*-acetoxy-12,13-dihydroxymulin-9-en (mulin-12,13-dihydroxy-9-en-7*β*-yl acetate)	
**92**	14α-acetoxy-13α-hydroxymulin-11-en-20-oic acid monohydrate (mulin-14α-acetoxy-13α-hydroxy-11-en-20-oic acid monohydrate)	[60]

* Previously reported as natural product.

**Table 4:** Mulinane derivatives obtained through biotransformation.

No.	Biotransformed Mulinanes	References
50 *	16-hydroxy-mulin-11,13-dien-20-oic acid (mulin-16-hydroxy-11,13-dien-20-oic acid)	[51,56]
**93**	7*α*,16-dihydroxymulin-11,13-dien-20-oic acid (mulin-7α,16-dihydroxy-11,13-dien-20-oic acid)	
**94**	Mulin-11,13-dien-16,20-oic acid	[55]
**95**	7*α*,13*β*-dihydroxymulin-11-en-dien-20-oic acid (mulin-7α,13β-dihydroxy-11-en-dien-20-oic acid)	

* Previously reported as natural product.

**Table 5:** Pharmacological activities of mulinanes and azorellanes. MIC: Minimal Inhibitory Concentration; ZI: Zone of inhibition; LD_50_: Median Lethal Dose; %I: Percentage of Inhibition; IC_50_: Median Inhibitory Concentration; %MC: Percentage of Motility Cells; %LC: Percentage of Living Cells.

Activity	Study Model	Compound (Number)	Skeleton Type	Compound Origin	Biological Result	Positive Control	References
Antimicrobial	*Mycobacterium tuberculosis* H37Rv (ATCC 27294)	13*α*-hydroxyazorellane (**11**)	A	N	MIC = 20 µg/mL	Rifampin MIC = 0.125 µg/mL	[46]
		13*β*-hydroxyazorellane (**15**)	A	N	MIC = 12.5 µg/mL	Rifampin MIC = 0.062 µg/mL Ofloxacin MIC = 0.125 µg/mL	[57]
		Azorellanol (**9**)	A	N	MIC = 12.5 µg/mL		
		17-acetoxy-13*α*-hydroxyazorellane (**27**)	A	N	MIC = 12.5 µg/mL		
		7*β*-deacetylazorellanol (**16**)	A	N	MIC = 25 µg/mL		
		Azorellanone (**17**)	A	N	MIC = 12.5 µg/mL		
		Mulin-11,13-dien-20-oic acid (**6**)	M	M	MIC = 50 µg/mL		
		Mulinic acid (**1**)	M	N	MIC = 50 µg/mL		
		Mulinol (**8**)	M	N	MIC = 25 µg/mL		
		7*β*-acetoxy-12*α*,13*α*-dihydroxy-mulin-9-ene (**54**)	M	SS	MIC = 25 µg/mL		
		13*α*-hydroxy-mulin-11-en-20-oic-acid methyl ester (**56**)	M	SS	MIC = 12.5 µg/mL		
		Mulin-11,13-dien-20-oic acid methyl ester (**57**)	M	SS	MIC = 25 µg/mL		
		Mulinenic acid methyl ester (60)	M	SS	MIC = 12.5 µg/mL		
		13*α* -hydroxy-mulin-11-en-20-oic-acid ethyl ester (**61**)	M	SS	MIC = 25 µg/mL	Rifampin MIC = 0.062 µg/mL Ofloxacin MIC = 0.125 µg/mL	[59]
		13*α* -hydroxy-mulin-11-en-20-oic-acid *n*-propyl ester (**62**)	M	SS	MIC = 25 µg/mL		
		Mulin-11,13-dien-20-oic acid ethyl ester (**63**)	M	SS	MIC = 25 µg/mL		
		Mulin-11,13-dien-20-oic acid *n*-propyl ester (**64**)	M	SS	MIC = 25 µg/mL		
		Mulin-11,13-dien-20-oic acid *n*-butyl ester (**65**)	M	SS	MIC = 25 µg/mL		
		Mulin-11,13-dien-20-oic acid *iso*-propyl ester (**66**)	M	SS	MIC = 50 µg/mL		
		Mulin-11,13-dien-20-oic acid *iso*-butyl ester (**67**)	M	SS	MIC = 50 µg/mL		
		Mulin-11,13-dien-20-oic acid *sec*-butyl ester (**68**)	M	SS	MIC = 50 µg/mL		
		17-acetoxy-mulinic acid *n*-propyl ester (**69**)	M	SS	MIC = 50 µg/mL		
		17-acetoxy-mulinic acid *iso*-butyl ester (**70**)	M	SS	MIC = 50 µg/mL		
		17-acetoxy-mulin-9(11),13(14)-dien-20-oic acid *iso*-propyl ester (**72**)	M	SS	MIC = 50 µg/mL		
		17-acetoxy-mulin-9(11),13(14)-dien-20-oic acid *sec*-butyl ester (**73**)	M	SS	MIC = 50 µg/mL		
		Isomulinic acid *n*-propyl ester (**74**)	M	SS	MIC = 25 µg/mL		
		Isomulinic acid *n*-butyl ester (**75**)	M	SS	MIC = 25 µg/mL		
		Isomulinic acid *iso*-propyl ester (**76**)	M	SS	MIC = 50 µg/mL		
		Isomulinic acid *iso*-butyl ester (**77**)	M	SS	MIC = 50 µg/mL		
	*M. tuberculosis* clinical isolate (MDR)	13*β*-hydroxyazorellane (**15**)	M	N	MIC = 25 µg/mL	Ofloxacin MIC = 0.250 µg/mL	[57]
		Azorellanol (**9**)	A	N	MIC = 12.5 µg/mL		
		17-acetoxy-13*α*-hydroxyazorellane (**27**)	A	N	MIC = 12.5 µg/mL		
		7*β*-deacetylazorellanol (**16**)	A	N	MIC = 25 µg/mL		
		Azorellanone (**17**)	A	N	MIC = 25 µg/mL		
		13*β*-epiazorellanol (**24**)	A	N	MIC = 50 µg/mL		
		mulin-13*α*-hydroxy-11-en-20-oic acid (**5**)	M	N	MIC = 50 µg/mL		
		Mulin-11,13-dien-20-oic acid (**6**)	M	N	MIC = 25 µg/mL		
		13*α*,14*α*-dihydroxy-mulin-11-en-20-oic acid (**18**)	M	N	MIC = 50 µg/mL		
		Mulinic acid (**1**)	M	N	MIC = 25 µg/mL		
		17-acetoxymulinic acid (**3**)	M	N	MIC = 50 µg/mL		
		Mulinol (**8**)	M	N	MIC = 12.5 µg/mL		
		7*β*-acetoxy-12*α*,13*α*-dihydroxy-mulin-9-ene (**54**)	M	SS	MIC = 25 µg/mL		
		7-oxo-mulin-9,12-diene (**55**)	M	SS	MIC = 50 µg/mL		
		13*α*-hydroxy-mulin-11-en-20-oic-acid methyl ester (**56**)	M	SS	MIC = 12.5 µg/mL		
		Mulin-11,13-dien-20-oic acid methyl ester (**57**)	M	SS	MIC = 12.5 µg/mL		
		Mulin-20-oic acid (**58**)	M	SS	MIC = 50 µg/mL		
		17-acetoxy-mulinic acid methyl ester (**59**)	M	SS	MIC = 50 µg/mL		
		Mulinenic acid methyl ester (**60**)	M	SS	MIC = 12.5 µg/mL		
		13*α*-hydroxy-mulin-11-en-20-oic-acid ethyl ester (**61**)	M	SS	MIC = 12.5 µg/mL	Ofloxacin MIC = 0.250 µg/mL	[59]
		13*α*-hydroxy-mulin-11-en-20-oic-acid *n*-propyl ester (**62**)	M	SS	MIC = 6.25 µg/mL		
		Mulin-11,13-dien-20-oic acid ethyl ester (**63**)	M	SS	MIC = 12.5 µg/mL		
		Mulin-11,13-dien-20-oic acid *n*-propyl ester (**64**)	M	SS	MIC = 12.5 µg/mL		
		Mulin-11,13-dien-20-oic acid *n*-butyl ester (**65**)	M	SS	MIC = 12.5 µg/mL		
		Mulin-11,13-dien-20-oic acid *iso*-propyl ester (**66**)	M	SS	MIC = 25 µg/mL		
		Mulin-11,13-dien-20-oic acid *iso*-butyl ester (**67**)	M	SS	MIC = 25 µg/mL		
		Mulin-11,13-dien-20-oic acid *sec*-butyl ester (**68**)	M	SS	MIC = 25 µg/mL		
		17-acetoxy-mulinic acid *n*-propyl ester (**69**)	M	SS	MIC = 12.5 µg/mL		
		17-acetoxy-mulinic acid *iso*-butyl ester (**70**)	M	SS	MIC = 25 µg/mL		
		17-acetoxy-mulin-9(11),13(14)-dien-20-oic acid *n*-propyl ester (**71**)	M	SS	MIC = 50 µg/mL		
		17-acetoxy-mulin-9(11),13(14)-dien-20-oic acid *iso*-propyl ester (**72**)	M	SS	MIC = 25 µg/mL		
		17-acetoxy-mulin-9(11),13(14)-dien-20-oic acid *sec*-butyl ester (**73**)	M	SS	MIC = 25 µg/mL		
		Isomulinic acid *n*-propyl ester (**74**)	M	SS	MIC = 6.25 µg/mL		
		Isomulinic acid *n*-butyl ester (**75**)	M	SS	MIC = 6.25 µg/mL		
		Isomulinic acid *iso*-propyl ester (**76**)	M	SS	MIC = 12.5 µg/mL		
		Isomulinic acid *iso*-butyl ester (**77**)	M	SS	MIC = 12.5 µg/mL		
		Isomulinic acid *sec*-butyl ester (**78**)	M	SS	MIC = 50 µg/mL		
	*M. smegmatis* (ATCC 14468)	7*β*-acetoxy-mulin-9,12-diene (**35**)	M	SS	MIC = 43.8 µg/mL	No data	[22]
	*Staphylococcus aureus* MSSA (ATCC 25923)	7*β*-acetoxy-mulin-9,12-diene (**35**)	M	SS	70 µg/dik: ZI = 15 mm	Penicillins/Streptomycin	[22]
	*S. aureus* clinical isolate MSSA	Mulin-12,14-dien-11-on-20-oic acid (**12**)	M	N	20 µg/disk: ZI = 8–12 mm	Vancomycin 30 µg/disk: ZI = 16–18 mm	[40]
		Mulin-12-ene-11,14-dion-20oic acid (**13**)	M	N	20 µg/disk: ZI = 8–12 mm		
	*S. aureus* clinical isolate MRSA	Mulin-12,14-dien-11-on-20-oic acid (**12**)	M	N	20 µg/disk: ZI = 8–12 mm		
		Mulin-12-ene-11,14-dion-20-oic acid (**13**)	M	N	20 µg/disk ZI = 8–12 mm		
	*S. aureus* clinical isolate MDR	7*β*-acetoxy-mulin-9,12-diene (**35**)	M	SS	70 µg/disk: ZI = 13 mm	Penicillin/Streptomycin	[22]
	*Escherichia coli* (ATCC BAS-849)	Mulin-12,14-dien-11-on-20-oic acid (**12**)	M	N	20 µg/disk ZI = 8–12 mm	Cefoxitin 30 µg/dik: ZI = 16–18 mm	[40]
		Mulin-12-ene-11,14-dion-20-oic acid (**13**)	M	N	20 µg/disk: ZI = 8–12 mm		
	*Enterococcus faecium* clinical isolate Vancomycin resistant	Mulin-12,14-dien-11-on-20-oic acid (**12**)	M	N	20 µg/disk: ZI = 8–12 mm	Bacitracin 10 µg/disk: ZI = 16–18 mm	
		Mulin-12-ene-11,14-dion-20-oic acid (**13**)	M	N	20 µg/disk: ZI = 8–12 mm		
Antiprotozoal	*Trichomonas vaginalis* trophozoite as (Ant-1 strain)	13*β*-hydroxyazorellane (**15**)	A	N	LD_50_ = 100 µM	Metronidazole LD_50_ = 6.6 µM	[35]
		13*α*-hydroxyazorellane (**11**)	A	N	LD_50_ = 119 µM		
		Azorellanol (**9**)	A	N	LD_50_ = 40.5 µM		
	*Toxoplasma gondii* trophozoites	7*β*-deacetylazorellanol (**16**)	A	N	LD_50_ = 54 µM	Clindamycin LD_50_ = 84 µM	[41]
		Azorellanol (**9**)	A	N	LD_50_ = 42 µM		
	*Trypanosoma cruzi* strains Tula-huen, SPA-14 and CL Brener clone	Mulin-11,3-dien-20-oic acid (**6**)	M	N	%I = 92–98.4 (IC_50_ = 41–87 µM) to 10 µM	Gentian violet 1 µM	[21]
		Azorellanol (**9**)	A	N	%I = 88.4–99IC_50_ = 20–84 µM to 10 µM		
	*Plasmodium berghei* (NK 65)	20-hydroxymulin-11,13-dienyl acetate (21)	M	N	20 mg/kg/day: %I = 29 to	Chloroquine 5 mg/kg/day IC_50_ = 2.5 mg/kg/day	[29]
		13*α*,14*α*-dihydroxymulin-11-en-20-oic acid (18)	M	N	20 mg/kg/day:%I = 42 to		
		17-acetoxymulin-11,13-dien-20-oic acid (**18**)	M	N	20 mg/kg/day:%I = 60 to		
Spermicidal/Spermatostatic	Human sperm, motile and living cells	Azorellanone (**17**)	A	N	3 mM: %MC = 41% 3 mM: %LC = 57	0.5% ethyl acetate (vol/vol)	[68]
	Human sperm, motile and living cells	Mulinonic acid (**5**)	M	N	%MC = 32% LC = 84	No data	[31]
	Human sperm, motile and living cells	Azorellan-17,13-(*β*)olide (azorellolide) (**22**)	A	N	%MC = 34% LC = 82	No data	
Cytotoxic	Cancer cell line MCF-7 (ATCC, Mana-sas VA, UA),	Azorellanol (**9**)	A	N	IC_50_ = 25.64 µM	Doxorubicin IC_50_ = 5.52 µM	[28]
		Mulin-11,13-dien-20-oic acid (**6**)	M	N	IC_50_ < 100 µM		
		7*β*-deacetylazorellanol (**16**)	A	N	IC_50_ < 100 µM		
		13*β*-epiazorellanol (**24**)	A	N	IC_50_ < 100 µM		
		7*β*-acetoxy-mulin-9,12-diene (**35**)	M	N	IC_50_ < 100 µM		
Anti-inflammatory	In vivo assay, arachidonic acid model	Azorellanol (**9**)	A	N	6.3 x 10^-6^ mol/ear: 38.6% anti-inflammatory effect	Nimesulide 3.2 x 10^-6^ mol/ear: 48.8% anti-inflammatory effect. Indo-methacin 1.4 x 10^-6^ mol/ear: 28.0% anti-inflammatory effect	[42]
	In vivo assay, 12-deoxy-phorbol 13 tetra-decanoate model	Azorellanol (**9**)	A	N	15.0 x 10^-7^ mol/ear: 70.8% anti-inflammatory effect	Indomethacin 1.4 x 10^-6^ mol/ear: 81.8% anti-inflammatory effect	
		7*β*-deacetylazorellanol (**16**)	A	N	2.6 x 10^-6^ mol/ear: 79.0% anti-inflammatory effect		
	anti-NF-kB	Azorellanol (**9**)	A	N	Inhibition at 25 mg	No data	[44]
		7*β*-deacetylazorellanol (**16**)	A	N	Inhibition at 25 mg		
		13*β*-hydroxyazorellane (**15**)	A	N	Inhibition at 25 mg		
Analgesic	In vivo assay, Acetic acid model	Azorellanol (**9**)	A	N	11.0 x 10^-5^ mol/kg: 50.7% of analgesic effect	Sodium naproxen 4.9x10^-5^ mol/kg: 70.0% of analgesic effect	[42]
		7*β*-deacetylazorellanol (**16**)	A	N	5.7 x 10^-5^ mol/kg: 53.4% of analgesic effect		
		Azorellanone (**17**)	A	N	10.0 x 10^-5^ mol/kg: 59.0% of analgesic effect		
Antihyperglycemic	In vivo assay Strepto-zotocin-induced diabetic	Mulinolic acid (**5**)	M	N	180 mg/mL: 48% reduction of glucose	Chlorpropamide 5 mg/mL: 50.3% reduction of glucose	[23]
		Azorellanol (**9**)	A	N	180 mg/mL: 49% reduction of glucose		
Antiulcer	In vivo assay HCl/EtOH-induced injury model	Mulin-11,13-dien-18-acetoxy-16,20-dioic acid (**31**)	M	N	20 mg/kg: 73% reduction of gastric injury	Lansoprazole 20 mg/kg: 78% reduction of gastric injury	[24]
		Azorellanol (**9**)	A	N	20 mg/kg: 71% reduction of gastric injury		
		13*β*-hydroxyazorellane (**15**)	A	N	20 mg/kg: 69% reduction of gastric injury		
		13*β*-hydroxymulinane (**28**)	M	N	20 mg/kg: 59% reduction of gastric injury		
		Mulin-11,13-dien-20-ol (**29**)	M	N	20 mg/kg: 26% reduction of gastric injury		
		Mulin-11,13-dien-20-oic acid (**6**)	M	N	20 mg/kg: 39% reduction of gastric injury		
		13*α*-hydroxyazorellane (**11**)	A	N	20 mg/kg: 56% reduction of gastric injury		
		Mulinolic acid (**5**)	M	N	20 mg/kg: 55% reduction of gastric injury		
	In vivo assay HCl/EtOH-induced injury model	Mulin-11,13-dien-20-oic acid (**6**)	M	N	ED_50_ = 55 mg/kg	Lansoprazole 20 mg/kg	[36]
	In vivo assay HCl/EtOH-induced injury model	16-hydroxy-mulin-11,13-dien-20-oic acid (**50**)	M	SS	20 mg/kg: 59% reduction of gastric injury	Lansoprazole 20 mg/kg: 57% reduction of gastric injury	[51]
		7*α*,16-dihydroxymulin-11,13-dien-20-oic acid (**93**)	M	SS	20 mg/kg: 69% reduction of gastric injury		
		16-acetoxymulin-11,13-dien-20-oic acid (**82**)	M	SS	20 mg/kg: 43% reduction of gastric injury		
		7*α*,16-diacetoxymulin-11,13-dien-20-oic acid (**81**)	M	SS	20 mg/kg: 48% reduction of gastric injury		
		7*α*,16-dihydroxymulin-11,13-dien-20-oic acid methyl ester (**85**)	M	SS	20 mg/kg: 36% reduction of gastric injury		
Anti-Alzheimer.	Inhibition of the enzyme acetyl-choline-esterase	Mulinolic acid (**5**)	M	N	IC_50_ = 200 µg/mL (630 µM)	Galanthamine IC_50_ = 1.1 µg/mL (3.0 µM)	[69]
	Inhibition of the enzyme acetyl-choline-esterase	Mulin-11,13-dien-20-oic acid (**6**)	M	N	IC_50_ = 180 µg/mL (580 µM)		

N: Natural; SS: Semisynthetic.
